# Thermal Hazard Characteristics of Unsaturated Polyester Resin Mixed with Hardeners

**DOI:** 10.3390/polym13040522

**Published:** 2021-02-10

**Authors:** Kewei Ren, Yunting Tsai

**Affiliations:** School of Chemical Engineering and Technology, Xi’an Jiaotong University, Xi’an 710049, China; renkewei@stu.xjtu.edu.cn

**Keywords:** curing reaction, polymerization, thermal runaway reaction, safer operating condition, explosion

## Abstract

Unsaturated polyester resin (UP) is a critical polymer material in applications of many fields, such as the chemical industry, military, and architecture. For improving the mechanical properties, some hardeners, such as methyl ethyl ketone peroxide (MEKPO) or tert-butyl peroxy-2-ethylhexanoate (TBPO), can trigger the curing reaction in UP polymerization, which causes that UP changes the structure from monomer to polymer. However, polymerization is a strong exothermic reaction, which can increase the risk of thermal runaway reaction in UP. Therefore, the mechanisms and characteristics in the thermal runaway reaction of UP mixed with hardeners should be studied for preventing and controlling UP explosion. The thermal hazards of UP mixed with hardeners were determined by thermogravimetric analyzer (TGA) and differential scanning calorimetry (DSC) analysis. According to the results, UP mixed with MEKPO exhibited a more violent mass loss and exothermic reaction than UP mixed with TBPO. Furthermore, the thermal runaway reactions of UP mixed with MEKPO or TBPO with different mixing proportions of 1:1, 3:1, and 5:1 were determined. Irrespective of MEKPO or TBPO, the mixing proportions of 3:1 exhibited a high onset temperature and low enthalpy of curing reaction (Δ*H*_exo_). This demonstrated that this proportion was safer during UP polymerization. The results of this study can provide useful information for preventing UP explosion and developing polymerization technology.

## 1. Introduction

Unsaturated polyester resin (UP), which is a critical polymer, is widely applied in many fields, such as the chemical industry, military, and architecture. Furthermore, China has the largest production of UP in the world. For the applications in many fields and better mechanical properties, hardeners are used to carry out the curing reaction for UP polymerization, thereby changing the properties of UP from liquid to solid. However, polymerization is a strong exothermic reaction. If heat generation is too fast or heat remove rate is slower than heat generation, a considerable heat can accumulate in the reaction system, thereby increasing the risk of the thermal runaway reaction, even fire and explosion [1,2,3,4,5,6]. Therefore, the thermal hazard of UP mixed with hardeners should be closely studied.

Many studies have investigated the material properties for UP after the curing reaction. Hong et al. explored the effects of the chain length of acid on the curing reaction of UP. The curing rate of UP became slower with the increase in the chain length of saturated aliphatic binary carboxylic acid, which can retard the curing process of UP [7]. Chu et al. investigated the properties of products in vinyl ester-unsaturated polyester resin with adipic acid, CaCO_3_, and polymer vinyl acetate after the curing reaction. Curing reaction was related to anti-shrinkage, and a decrease in the curing degree can increase the effects of anti-shrinkage [8]. Xu et al. reported that some promoters can shorten the curing time and temperature in the curing reaction [9]. Furthermore, the flame retardancy of UP mixed with flame retardants was explored. Bai et al. synthesized a sort of phosphorus-containing star-shaped flame retardant, which can considerably improve the flame retardancy of UP in both gas and condensed phases at the same time [10]. Zhao et al. designed a novel organophosphorus polymeric flame retardant, which was synthesized by hydroxyphenyl imino methyl phenol spirocyclic pentaerythritol diphosphonate, incorporated into the UP monomer [11]. Salasinska et al. developed several kinds of novel flame retardants for UP with high nitrogen content, but some of the retardants may affect the curing process [12]. Boulkadid et al. summarized the various methods for improving and optimizing the curing reaction of high-energy composite materials [13]. Bessa et al. investigated the curing kinetics of bisphenol A-based benzoxazine under non-isothermal and isoconversional kinetic methods by differential scanning calorimetry (DSC) technique [14].

In the past, the curing properties of UP were fully investigated. However, if human error, overfeeding, or equipment failure happen in the cross-linking polymerization of UP, considerable amounts of hardeners may enter into the polymerization system and react with UP, thereby increasing the risk of thermal hazards in UP mixed with hardeners [15]. Furthermore, the thermal hazard characteristics of UP mixed with hardeners were rarely explored, which can cause some faulty experiences in preventing and controlling the thermal runaway of UP, thereby resulting in more serious accidents [16,17]. In this study, critical thermal stability parameters, such as mass loss, mass derivative, onset temperature (*T*_0_), peak temperature (*T*_p_), and enthalpy of curing reaction (Δ*H*_exo_), were used to evaluate the thermal hazard of UP mixed with two commercial hardeners, methyl ethyl ketone peroxide (MEKPO) and tert-butyl peroxy-2-ethylhexanoate (TBPO), by thermogravimetric analyzer (TGA) and DSC analysis. This result can provide useful information for inherently safer design of UP during its use, transportation, storage, or deposit and for developments of polymerization technologies.

## 2. Materials and Methods

### 2.1. Samples

UP (6120-TA) with 30% styrene was provided by En Chuan Chemical Industry, Taichung, Taiwan. Methyl ethyl ketone peroxide (MEKPO) and tert-butyl peroxy-2-ethylhexanoate (TBPO), which were purchased from ACE Chemical Corporation, Taoyuan, Taiwan, were stored in sealed containers in a dry and ventilated environment, and the storage temperature should be lower than 4 °C. The mixing proportions of UP mixed with TBPO or MEKPO were 1:1, 3:1, and 5:1. For simplification, UP mixed with TBPO or MEKPO is termed UP-TBPO or UP-MEKPO, respectively.

### 2.2. Methods

TGA (Pyris1, PerkinElmer, Waltham, MA, USA) was used to investigate the mass loss and mass derivative of UP, UP-MEKPO, and UP-TBPO. The mass of pure UP was 3.0–3.5 mg. The heating rates were 10, 15, 20 and 25 °C/min, and the temperature range was 30–800 °C under a nitrogen atmosphere at 20 mL/min. DSC (DSC 821e, Mettler-Toledo, Greifensee, Switzerland) was used to observe the exothermic reaction of UP, UP-MEKPO, and UP-TBPO, which can obtain critical thermodynamic parameters, such as *T*_0_, *T*_p_, and Δ*H*_exo_. The masses of UP mixed with hardeners for 1:1, 3:1, and 5:1 were 3.5–4.0, 4.0–5.5, and 6.0–6.5 mg, respectively. The heating rates were 2, 4, 8, and 16 °C/min, and the temperature range was 30–300 °C under a nitrogen atmosphere at 20 mL/min. Moreover, oven heating was used to carry out the curing reaction for UP-TBPO or UP-MEKPO at 150 °C and 20 min.

### 2.3. Kinetic Analysis

Kinetic studies were conducted by the Kissinger method [18,19,20,21], as given in Equation (1):

(1) $$\frac{d\alpha }{dT}=\frac{A}{\beta }\exp\left(-\frac{E_{a}}{RT}\right)f\left(\alpha \right)$$

where α is degree of conversion, *T* is absolute temperature, *β* is heating rate, R is gas constant [8.314 J/(mol·K)], A is pre-exponential factor, *E*_a_ is activation energy, *f*(α) is mechanism function.

When *T* is equal to *T*_p_, Equation (2) can be obtained by taking natural logarithm of Equation (1).

(2) $$ln\left(\frac{\beta }{T_{p}^{2}}\right)=\ln\left(\frac{\mathrm{AR}}{E_{a}}\right)+\ln{\left[\frac{-df\left(\alpha \right)}{d\alpha }\right]}_{\alpha_{p}}-\frac{E_{a}}{RT_{p}}.$$

Therefore, the slope of plot of $\ln\left(\frac{\beta }{T_{p}^{2}}\right)$ versus $\left(\frac{1}{T_{p}}\right)$ can be used to determine the apparent activation energy (*E*_a_), and the mechanism function does not need to be considered [20,21].

## 3. Results and Discussion

**Figure 1:**
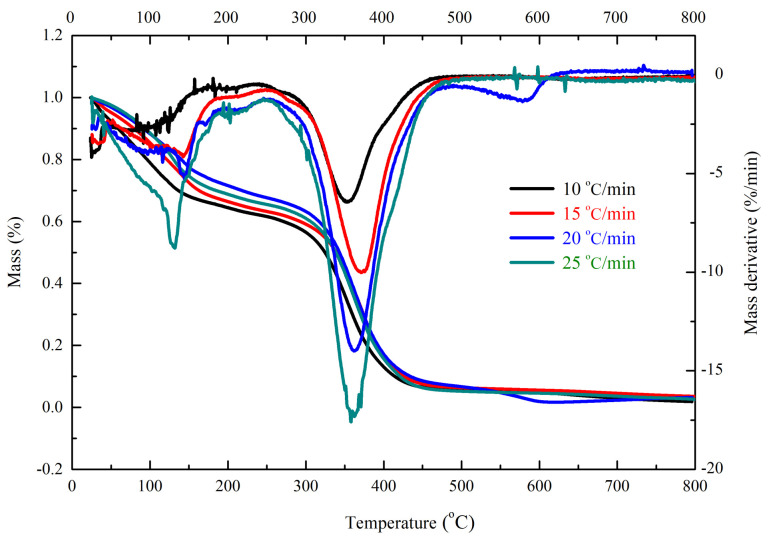
Mass and mass derivative curves of pure unsaturated polyester resin (UP) at heating rates of 10, 15, 20, and 25 °C/min.

Figure 1 displays the mass and mass derivative curves for pure UP at the heating rates of 10, 15, 20, and 25 °C/min. We conducted three replications for thermogravimetry (TG) experiments. The mass of UP gradually declined with the increase in the temperature. Two obvious mass loss stages were observed in the thermal decomposition of UP at the heating rates of 10, 15, and 25 °C/min. The first peak occurred in the temperature range of 30–200 °C and second one occurred in the range of 220–500 °C. Because 30% styrene existed in UP, the first curve was styrene volatilization and second one was UP decomposition. However, UP exhibited three mass loss stages at the heating rates of 20 °C/min. The first stage was the volatilization of styrene, the second one was the UP decomposition, and third one was the UP residues decomposition. Because the volatilization of styrene and UP residues decomposition depended on the content levels of styrene and impurity, the thermal curves of the first and third stages may have slight differences in the TG analysis. Furthermore, the second stage of UP decomposition exhibited a great regularity and reproducibility with increasing the heating rates, which corresponded to typical TG results. Therefore, the results are reliable. Table 1 displays the related characteristic parameters for UP tested by TGA.

**Figure 2:**
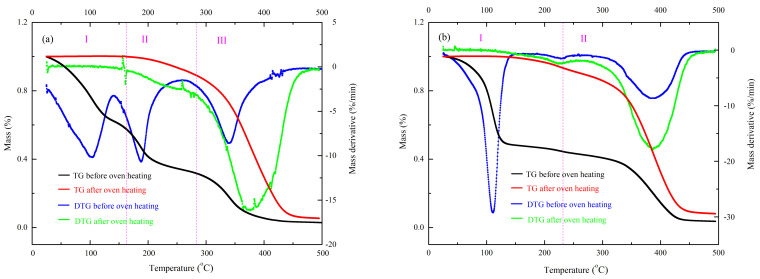
Mass and mass derivative curves of (**a**) unsaturated polyester resin-methyl ethyl ketone peroxide (UP-MEKPO) and (**b**) unsaturated polyester resin-tert-butyl peroxy-2-ethylhexanoate (UP-TBPO) after and before oven heating.

Figure 2 displays the mass and mass derivative curves of UP-MEKPO and UP-TBPO after and before oven heating. Three mass loss stages existed in UP-MEKPO before oven heating. The first peak was also styrene volatilization. Because the second and third peaks were close to the decomposition temperature of MEKPO and UP, respectively, these two peaks were individually MEKPO and UP decomposition [22]. However, only one mass loss curve existed in UP-MEKPO after oven heating. Because the decomposition temperature of styrene was lower than 200 °C and a curing reaction of UP-MEKPO occurred after oven heating, the first and two curves cannot be observed. Moreover, the mass loss of third curves after oven heating was higher than before one, which indicated that the UP and the product of UP-MEKPO were simultaneously decomposed in this stage. The mechanisms of curing reaction in UP-MEKPO were determined.

Two mass loss stages were observed in UP-TBPO before oven heating and only one existed in after one. Because the decomposition temperatures of styrene and TBPO were close, the combination curves of them occurred in the first stage, and UP decomposition occurred in the second one [23]. Furthermore, because a curing reaction occurred, the curve of UP-TBPO after oven heating was due to the UP decomposition combined with the curing product decomposition. This condition was similar to the results of UP-MEKPO. Table 2 displays the related characteristic parameters for UP-MEKPO and UP-TBPO after and before oven heating tested by TGA.

**Figure 3:**
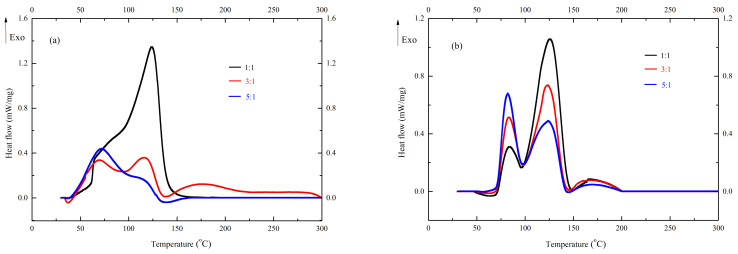
Heat flow versus temperature for (**a**) UP-MEKPO and (**b**) UP-TBPO with different mixing proportions of 1:1, 3:1, and 5:1 at a heating rate of 4 °C/min.

Figure 3a displays the heat flow versus temperature for UP-MEKPO with different mixing proportions of 1:1, 3:1, and 5:1 at a heating rate of 4 °C/min. DSC results were used to observe the exothermic characteristics of the curing reaction of UP mixed with hardeners. Two exothermic curves can be observed in UP-MEKPO. Because the thermal decomposition temperature of the curing products in UP-MEKPO was beyond 200 °C, the product decomposition could be excluded. An increase in the UP levels can increase the strength of the first peak. Therefore, the first stage was the heat release in the curing reaction of UP. As seen in Figure 3a, the temperature of the second curves was close to MEKPO decomposition, and an increase in MEKPO levels can increase the heat release, so this stage was the thermal decomposition of MEKPO. In addition, the study by Chi et al. [24] proposed that MEKPO decomposed at 42 °C, but the trace thermal release existed in this stage. The main thermal release curve of MEKPO initialed at 83 °C and its peak temperature was 130 °C, which corresponded to the second stage of UP-MEKPO decomposition. Therefore, the curing mechanisms of UP-MEKPO were determined. The first and second stages were the curing reaction of UP and MEKPO decomposition, respectively. Moreover, when the mixing proportion of UP-MEKPO was 1:1, a low *T*_0_ not only existed but also the Δ*H*_exo_ was far stronger than the proportions of 3:1 and 5:1, which indicated that the proportion of 1:1 had a high risk of runaway reaction. Moreover, a similar Δ*H*_exo_ existed in the proportions of 3:1 and 5:1, but a higher *T*_0_ existed in 3:1. This demonstrated that the mixing proportion of 3:1 exhibited a safer condition for the curing reaction of UP-MEKPO. When the proportion of UP-MEKPO was greater than the safe proportion, the heat release of the curing reaction also greatly increased, which may increase the risk of thermal hazards.

In Figure 3b, two exothermic curves also can be observed in UP-TBPO. For a similar condition of UP-MEKPO, an increase in the UP levels can increase the strength of the first peak, and an increase in the TBPO levels can increase the second one. The study by Tsai et al. [25] proposed that TBPO exhibited a single thermal release curve. The onset temperature of TBPO was close to 80 °C and its peak temperature was 125 °C, which corresponded to the second stage of UP-TBPO decomposition. Therefore, the first and second stages were the curing reaction of UP and TBPO decomposition, respectively. The curing mechanisms of UP-TBPO were determined as well. Moreover, the proportion of 3:1 also exhibited a higher *T*_0_ and a similar Δ*H*_exo_, compared to 1:1 and 5:1, and the proportion was a dangerous condition. Therefore, the proportion of 3:1 was also a safer condition for the curing reaction of UP-TBPO. Furthermore, the comparisons of exothermic characteristics between UP-MEKPO and UP-TBPO with different mixing proportions were determined. According to the results, UP-MEKPO exhibited a similar Δ*H*_exo_, compared to UP-TBPO in different mixing proportions, but a lower *T*_0_ than UP-TBPO. Therefore, UP-MEKPO had higher risks of a runaway reaction than UP-TBPO. Due to reasons such as human error, overfeeding, or equipment failure, excessive amounts of peroxide added to the system should be avoided.

**Figure 4:**
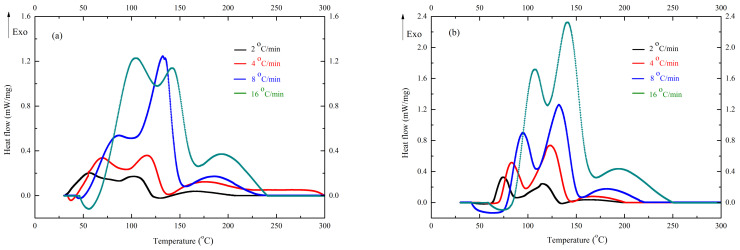
Differential scanning calorimetry (DSC) results of (**a**) UP-MEKPO and (**b**) UP-TBPO with different heating rates of 2, 4, 8, and 16 °C/min.

After confirmations of the safer conditions for UP-TBPO and UP-MEKPO, Figure 4 displays the DSC results of UP-TBPO and UP-MEKPO with different heating rates of 2, 4, 8, and 16 °C/min, which indicated the relationship between exothermic characteristics and heating rates. The *T*_0_ range of UP-MEKPO with different heating rates was 40.60–67.74 °C, *T*_p1_ was 57.02–103.10 °C, *T*_p2_ was 103.27–141.39 °C, and Δ*H*_exo_ was 280.34–401.53 J/g. The *T*_0_ range of UP-TBPO with different heating rates was 63.94–108.71 °C, *T*_p1_ was 74.35–106.54 °C, *T*_p2_ was 114.62–140.88 °C, and Δ*H*_exo_ was 355.57–375.79 J/g. Moreover, an increase in the heating rate can shift the exothermic curve of UP-TBPO and UP-MEKPO to a higher temperature, which exhibited a typical DSC experimental result. Therefore, the data of this study are reliable.

**Figure 5:**
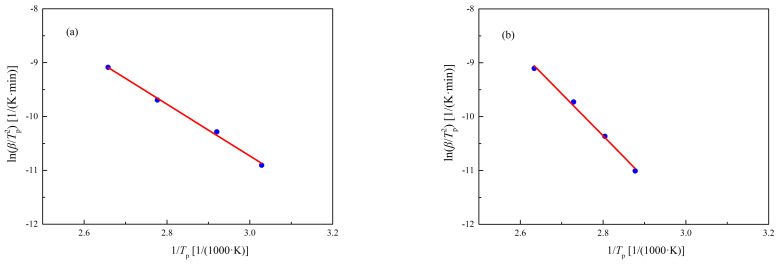
Plot of $\ln\left(\frac{\beta }{T_{p}^{2}}\right)$ versus $\left(\frac{1}{T_{P}}\right)$ of (**a**) UP-MEKPO and (**b**) UP-TBPO. Here, *β* is heating rate and *T*_p_ is peak temperature.

The *E*_a_ values of UP-MEKPO and UP-TBPO were calculated by the Kissinger method. Figure 5 displays the linear regression of the plot of $\ln\left(\frac{\beta }{T_{P}^{2}}\right)$ versus $\left(\frac{1}{T_{P}}\right)$, which indicated that the slope was *E*_a_. The *E*_a_ values of UP-MEKPO and UP-TBPO were 39.90 and 65.04 kJ/mol, respectively. Because *E*_a_ means the minimum energy for triggering an initial chemical reaction [26], the risk of thermal runaway reaction was higher in UP-MEKPO, which corresponded to the experimental results.

## 4. Conclusions

This study revealed the mass loss and exothermic characteristics for the curing reactions of UP-TBPO and UP-MEKPO, which can determine the explosion risk of UP during the polymerization. The critical findings are summarized as follows:

The thermal stability of UP and UP mixed with hardeners before or after the curing reaction was confirmed by TGA and DSC results. Because *T*_0_ can indicate the difficulty degree of the exothermic reaction and Δ*H*_exo_ can indicate the severity of the runaway reaction, the explosion risk can be determined by these two parameters. According to the results, both UP-MEKPO or UP-TBPO had a high *T*_0_ and low Δ*H*_exo_ at the mixing proportion of 3:1. Therefore, this proportion was a safer condition in the curing reaction of UP-MEKPO or UP-TBPO. At same mixing proportion, UP-TBPO exhibited a higher *T*_0_ and lower Δ*H*_exo_. Therefore, for considerations of inherently safer design, TBPO was deemed suitable as a hardener for the curing reaction of UP. This study can provide useful information for preventing and controlling the runaway reaction of UP mixed with hardeners and for development of novel hardeners in the future.

## Figures and Tables

**Table 1:** Related characteristic parameters for pure unsaturated polyester resin (UP) tested by thermogravimetric analyzer (TGA).

*β* (°C/min)	*T*_01_ (°C)	*T*_p1_ (°C)	Mass Loss (wt%)	*T*_02_ (°C)	*T*_p2_ (°C)	Mass Loss (wt%)	*T*_03_ (°C)	*T*_p3_ (°C)	Mass Loss (wt%)	Char Residue (wt%)
10	30.1	128.2	36.3	263.3	354.5	61.8	-	-	-	1.9
15	33.4	128.5	34.3	264.2	371.1	62.2	-	-	-	3.5
20	37.2	131.7	28.9	286.2	362.2	65.8	510.6	583.8	2.1	3.2
25	40.6	141.3	30.1	289.6	363.9	67.3	-	-	-	2.6

Abbreviation: *β* is heating rate, *T*_0_ is onset temperature, *T*_p_ is peak temperature, and the numbers (typed in a smaller font) indicate mass loss stages.

**Table 2:** Related characteristic parameters for unsaturated polyester resin-methyl ethyl ketone peroxide (UP-MEKPO) and unsaturated polyester resin-tert-butyl peroxy-2-ethylhexanoate (UP-TBPO) after and before oven heating tested by TGA.

Sample	*T*_01_ (°C)	*T*_p1_ (°C)	Mass Loss (wt%)	*T*_02_ (°C)	*T*_p2_ (°C)	Mass Loss (wt%)	*T*_03_ (°C)	*T*_p3_ (°C)	Mass Loss (wt%)	Char Residue (wt%)
UP + MEKPO (before oven heating)	37.2	104.1	37.8	149.1	189.6	29.2	285.6	340.3	31.6	1.4
UP + MEKPO (after oven heating)	235.9	376.4	94.7	-	-	-	-	-	-	5.3
UP + TBPO (before oven heating)	56.8	111.1	53.7	235.9	383.9	42.7	-	-	-	3.6
UP + TBPO (after oven heating)	213.4	396.5	91.9	-	-	-	-	-	-	8.1

## Data Availability

The data presented in this study are available on request from the corresponding author.
